# Communication interventions to improve adherence to infection control precautions: a randomised crossover trial

**DOI:** 10.1186/1471-2334-13-72

**Published:** 2013-02-06

**Authors:** Mei-Sing Ong, Farah Magrabi, Jeffrey Post, Sarah Morris, Johanna Westbrook, Wayne Wobcke, Ross Calcroft, Enrico Coiera

**Affiliations:** 1 Centre for Health Informatics, University of New South Wales, Sydney, Australia; 2 Department of Infectious Diseases, Prince of Wales Hospital, Sydney, Australia; 3 Prince of Wales Clinical School, University of New South Wales, Sydney, Australia; 4 Department of Medical Imaging, Prince of Wales Hospital, Sydney, Australia; 5 Centre for Health Systems and Safety Research, University of New South Wales, Sydney, Australia; 6 School of Computer Science and Engineering, University of New South Wales, Sydney, Australia; 7 Intensive Care Unit, Liverpool Hospital, Sydney, Australia

## Abstract

**Background:**

Ineffective communication of infection control requirements during transitions of care is a potential cause of non-compliance with infection control precautions by healthcare personnel. In this study, interventions to enhance communication during inpatient transfers between wards and radiology were implemented, in the attempt to improve adherence to precautions during transfers.

**Methods:**

Two interventions were implemented, comprising (i) a pre-transfer checklist used by radiology porters to confirm a patient’s infectious status; (ii) a coloured cue to highlight written infectious status information in the transfer form. The effectiveness of the interventions in promoting adherence to standard precautions by radiology porters when transporting infectious patients was evaluated using a randomised crossover trial at a teaching hospital in Australia.

**Results:**

300 transfers were observed over a period of 4 months. Compliance with infection control precautions in the intervention groups was significantly improved relative to the control group (p < 0.01). Adherence rate in the control group was 38%. Applying the coloured cue resulted in a compliance rate of 73%. The pre-transfer checklist intervention achieved a comparable compliance rate of 71%. When both interventions were applied, a compliance rate of 74% was attained. Acceptability of the coloured cue was high, but adherence to the checklist was low (40%).

**Conclusions:**

Simple measures to enhance communication through the provision of a checklist and the use a coloured cue brought about significant improvement in compliance with infection control precautions by transport personnel during inpatient transfers. The study underscores the importance of effective communication in ensuring compliance with infection control precautions during transitions of care.

## Background

Hospital acquired infection (HAI) constitutes a major public health problem worldwide. Approximately one in ten hospitalized patients has an infection acquired after admission [1]. The economic burden imposed by HAIs is considerable, with infected patients remaining in hospital 2.5 times longer on average and incurring costs almost three times higher than uninfected patients [2].

One of the most common modes HAI-associated pathogens are transmitted is through the contaminated hands of healthcare personnel [3]. Several studies have shown that patient-to-patient transmission through the hands of healthcare personnel is a major contributor to the spread of multidrug-resistant organisms, such as *methicillin-resistant Staphylococcus aureus* (MRSA) and *vancomycin-resistant enterococcus faecium* (VRE) [4-6]. Guidelines for preventing HAI transmission have been established by the US Centers for Disease Control (CDC) [7]. Standard precautions include the use of personal protective equipment, the safe use and disposal of sharps, decontamination of equipment and environment, patient placement and linen and waste management.

Whilst evidence suggests that these guidelines are effective in reducing HAI transmission [8-11], adherence to infection control practices remains poor. A recent review showed that observed adherence to hand hygiene was 52% (range 27–86%), and glove compliance and the use of gown or other protective clothing was 62% (range 11–98%) and 57% (range 8–93%) respectively [12]. The primary reasons for non-compliance identified were availability of time and protective clothing, workload, perception of risk, lack of knowledge and forgetfulness [12-16].

A recent study of inpatient transfers to radiology showed that in more than 30% of transfers involving patients with MRSA or VRE, standard infection control precautions were not followed [17]. Observations of the transfer process further revealed that ineffective communication of a patient’s infectious status was a potential contributor to non-compliance. Whilst standard precautions were the recommended practice when handling patients with unknown infectious status, they were rarely practiced during transfer, unless the transport personnel were informed that a patient was infectious. Whilst the study did not evaluate the association between poor compliance and the actual rate of infection, the potential risk clearly exists. Several epidemiological studies have reported that the transfer of patients carrying pathogens contribute to the spread of multi-resistant bacteria, with the risk of acquiring an infection after transfer being more than two times higher than the risk of a non-transferred patient becoming infected on a given day [18-20]. One study found that the proportion of MRSA was four times higher in transferred patients, independent of markers of illness severity such as length of stay [18]. In another matched case control study, intrahospital transfer of patients to more than one ICU or to more than one floor throughout their hospital stay increased the risk for acquisition of VRE [20].

Despite evidence of the potential risks of HAI transmission through inpatient transfers, there is little research on this topic. The role of effective communication in promoting compliance with precautions during transitions of care also remains relatively unexplored.

In this study, we examined the effectiveness of two simple interventions to improve communication of infection control requirements during inpatient transport to radiology. The interventions comprised (i) a checklist to promote proactive communication; and (ii) a coloured cue to enhance the prominence of written information. We hypothesised that improved communication would enhance compliance with infection control precautions during transfers.

## Methods

### Settings and participants

**Figure 1:**
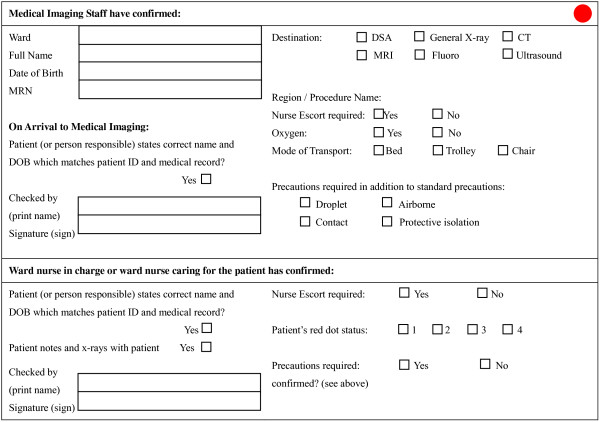
**Transfer form, with a coloured cue applied.** A red cue was applied to the transfer form to highlight the need for infection control precautions. A blue sticker was used to indicate the need for clinical escort.

The study was conducted at a 440-bed metropolitan teaching hospital with an average occupancy rate of 90%, and over 3000 staff members. Several hospital-wide infection control policies were in place at the time of the study. These included contact isolation and standard precautions (wearing of gloves, gown, and the practice of hand antisepsis) for suspected or known MRSA/VRE colonized or infected patients (Table 1). Gloves, gowns, sinks and alcohol-based disinfectant were conveniently located at every ward. Additionally, signage indicating isolation precaution was displayed on the door of the patient’s room. The primary tool for communicating transfer information was a transfer form, given to the porters by the radiology coordinator prior to the transfer. The form contained information about the patient, and any transport requirements including infection control precautions (Figure 1). Educational seminars were regularly held at the hospital to inform healthcare personnel of the best practices for infection control.

**Table 1:** **Hospital infection control practice when transferring patients requiring contact precautions **[21]

**Precaution**	**Protocol description**
**Standard precautions**	Standard precautions should be adhered to during patient transfer, including:
	• Hand antisepsis
	• Appropriate use of gloves and gowns
**Hand antisepsis**	Situations requiring hand antisepsis:
	• Before and after patient care procedures
	• Before and after direct patient contact
	• Before donning gloves and after removing gloves
	• After removing a gown
	• After touching inanimate objects that are likely to be contaminated
**Use of gloves**	Gloves must be worn during contact precautions, and must be changed and discarded:
	• After contact with a patient is complete and before care is provided to another
	• patient
	• Before touching environmental items and surfaces
	• Before or on leaving a patient’s room
	• Before writing in the medical notes, using the computer and moving or touching equipment
**Use of gowns**	Gowns must be worn on entering an isolation room during contact precautions, if contact with the patient or patient’s environment is likely, and removed before or immediately on exiting the room.

### Data collection

Radiology porters were shadowed unobtrusively by the researchers as they transferred inpatients to radiology. All radiology porters and transfers between radiology and inpatient wards (with the exception of emergency department and intensive care unit) were eligible for inclusion. Participation in the study was voluntary.

The primary outcome measure was the rate of compliance with infection control precautions by the porters when transferring patients between inpatient wards and radiology. Compliance was measured as a dichotomous all-or-none variable. Partial or full precautions taken prior to contact with patient were counted as compliance. When precautions were taken after patient contact, or when no precautions were observed, non-compliance was recorded. Full precautions included: wearing of gloves and gown and the practice of hand antisepsis (either by washing hands with soap and water, or by waterless antiseptic agent). The types of precautions taken were noted for each transfer. Secondary outcome measures included (1) adherence to the pre-transfer checklist; (2) any adverse effects caused by the interventions; and (3) the participants’ reactions to the interventions, assessed through informal interviews. Other data collected included the source of transfer (inpatient ward), and the timing of transfer.

Prior to study commencement, the porters were briefed individually by the radiology nursing manager on the nature of the interventions. The subjects were informed that the purpose of the study was to examine how the interventions would affect communication patterns during transfers. The true intent of this study was not revealed to the subjects.

Collection of data was performed by two researchers, covering transfers from morning to evening, Monday to Friday. A structured data collection tool was used (Additional file 1). Inter-rater reliability was performed by a second observer shadowing alongside the first for 10 transfers. Inter-rater reliability for determining compliance with infection control precautions was high (kappa = 0.99, 95% CI 0.98-1.00). All data collected was de-identified.

Approval to undertake the study was granted by the ethics committee of the hospital and the University of New South Wales. Written informed consent was obtained from all study participants. Informed consent from the patients was waived by the ethics committees.

### Interventions

**Figure 2:**
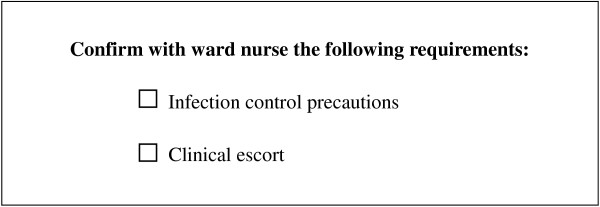
Checklist intervention.

Two interventions were developed to improve communication of infection control precautions. The first involved a pre-transfer checklist containing two items: (1) infection control precautions; and (2) clinical escort requirements (Figure 2). The latter was included to blind the participants of the true intent of the study. The checklist was given to the porter by the researcher at the beginning of a transfer. On patient collection, it was used by the porters to confirm with the ward nurse requirements for infection control precautions and clinical escort.

In the second intervention, a coloured cue was implemented (Figure 1). A red sticker was attached to the form to indicate that infection control precautions were required, and a blue sticker indicated that a clinical escort was required. Application of the sticker was performed by either the researcher or the radiology coordinator at the beginning of the transfer, out of the view of the porters.

**Figure 3:**
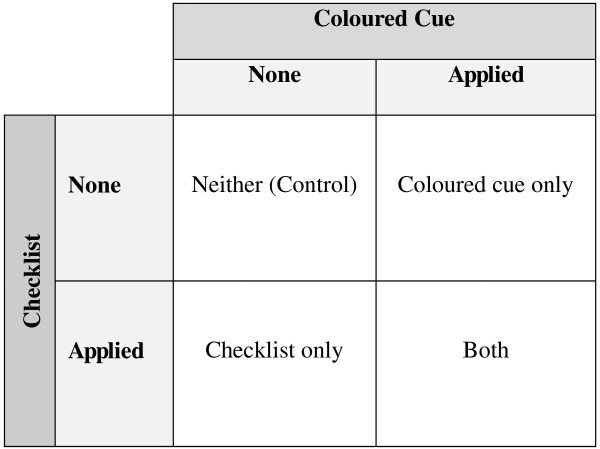
Study design using a 2x2 factorial design with two interventions.

A repeated measure crossover design was used for the study. There were 4 study arms: (1) pre-transfer checklist; (2) coloured cue; (3) both interventions; and (4) control arm without any interventions (Figure 3). Each porter was included in all study arms. Repeated observations were carried out for each porter in every study arm. The number of repeated measurements per porter was determined by a power analysis algorithm [22,23].

A computerized random-number generator was used to generate a random allocation sequence, with equal number for each study arm. Interventions were assigned to transfers based on this sequence. Since transfers were sometimes cancelled, randomisation alone was inadequate in ensuring balance between study arms. Minimisation was employed in the later stage of the study, where study arms with low numbers of observations were given weighted preference [24]. Observations were carried out until the minimum set of transfers per study arm was achieved.

### Statistical analysis

Comparisons of the rate of compliance with infection control precautions between study arms was carried out using *χ*^2^ test statistic. To adjust for the effects of within-subject correlations, the conventional *χ*^2^ test statistic was divided by a correction factor [25]. Comparisons of the average transport time between study arms were carried using t-statistics. Descriptive statistics were used to summarise transfer characteristics and to assess adherence to the pre-transfer checklist.

## Results

### Transfer demographics

The study was conducted over a period of four months (March 2010 – June 2010). In total, 11 porters were observed over 300 transfers. Of these, 179 (60%) transfers involved a patient infected with MRSA or VRE. The remaining patients were not known to be infectious. The total number of transfers to radiology from the wards over this period was about 8320, a daily average of 80. More than half the transfers were from the infectious diseases unit (14.3%), spinal injury unit (10.3%), coronary care unit (9.7%), renal ward (9.7%) and surgery (9.7%). The remainder were spread over 12 other specialties.

### Participant flow

**Figure 4:**
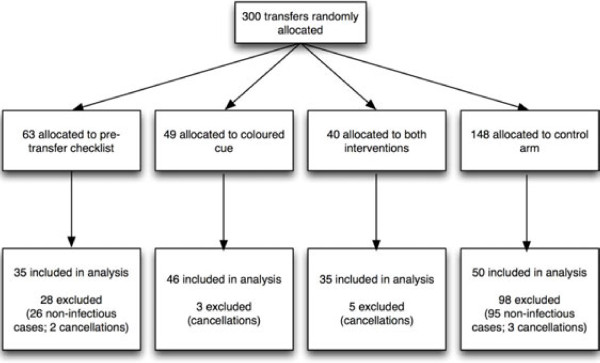
Participant flow.

There were five permanent radiology porters, all of whom were recruited for the study. One porter resigned from the hospital three weeks into the study and was replaced by five temporary porters from other departments, before a permanent replacement was finally recruited. Thus, the study data consisted of five subjects with the full set of repeated measures per study arm, partial data from the porter who resigned, and the temporary porters (Figure 4).

### Compliance with infection control precautions

Compliance with infection control precautions in all intervention groups was significantly improved relative to the control group (p < 0.01) (Table 2). In the control group, overall adherence (full or partial) to infection control precautions was observed in 38% of transfers involving an infectious patient. Applying a coloured cue on the transfer form resulted in a compliance rate of 73%. The pre-transfer checklist intervention achieved a comparable compliance rate of 71%. When both interventions were applied, a compliance rate of 74% was attained. The differences between intervention arms were not significant.

**Table 2:** Compliance with infection control precautions in each study arm

**Outcomes**	**Study arm**			
	**Checklist(n = 35)**	**Cue(n = 46)**	**Both(n = 35)**	**Control(n = 50)**
Compliance with precautions, full or partial	71%	73%	74%	38%
Adjusted χ^2^	7.05	9.52	7.19	-
P value	< 0.01	< 0.01	< 0.01	-
Compliance with specific precautions				
Hand antisepsis	14%	11%	9%	4%
Wearing of gloves	71%	74%	71%	38%
Wearing of gown	29%	41%	34%	28%
Full compliance (hand antisepsis, gloves, gown)	14%	11%	9%	4%
Adherence to pre-transfer checklist	40%	-	40%	-

### Compliance with the Pre-transfer checklist

The pre-transfer checklist was completed in 40% of cases. In most cases, the porters did not interact with the ward nurses. Rather, they relied on other visual cues within the ward to complete the checklist. Potential cues indicating the need for precautions included: warning poster on the ward, the patient was placed in isolation room, or ward nurses were wearing gloves and gowns when handling the patient. There was no significant difference in the compliance rate of standard precautions between the cases where the checklist was presented to the porters and subsequently completed by them (25%), and the cases where the checklist was not completed (28%) (p = 0.88).

### Adverse effects of interventions

Introduction of the interventions did not result in any observable adverse effects. Implementation of the pre-transfer checklist could have potentially introduced delay in the transfer process, due to the additional steps required to perform the checklist. This is unlikely as the checklist was performed when the ward nurses were signing the transfer form, and therefore did not interfere with the transfer workflow. Comparing the total transport time between the control group and the intervention groups using *t*-test statistics confirmed that there was no significant difference in the average time per transfer.

### Reactions to interventions

Whilst the pre-transfer checklist resulted in improved compliance with infection control precautions, compliance with the checklist itself was poor. Observations identified several barriers to the use of checklist. Firstly, ward nurses were often busy attending to patients during patient collection, therefore communication between porters and nurses was typically reduced to the bare essentials. Secondly, the checklist was not well-received by some porters. When presented with the checklist, one porter openly expressed: “I do the checks already, it’s part of my job, you don’t need to give me that.”

In contrast, the coloured cue gained good acceptance amongst both nurses and porters. Informal interviews with the porters and nurses showed that both groups perceived the intervention to be an effective visual reminder for infection control requirements.

## Discussion

The results of this study support the hypothesis that improving communication of infection control requirements may lead to better compliance with precautions during inpatient transfers. Introduction of a pre-transfer checklist promoted communication between porters and ward nurses, and therefore the porters were better informed of a patient’s infectious status. Using a coloured sticker to flag an infectious case was an effective means for cueing the porters to the importance of written information. In both interventions, the adherence rate to infection control precautions was significantly improved.

In this study, the researcher was actively involved in applying the interventions. In practice, both the checklist and coloured cue can be easily incorporated into existing workflow, without imposing additional workload. For example, simple software can be implemented so that the coloured cue is automatically inserted into the printed transfer form. The existing transfer form can also be extended to include a checklist for infection control precaution.

### Effectiveness of the pre-transfer checklist

Adherence to the checklist was lower than expected (40%). Observations showed that subjects mostly completed the checklist by inspecting visual cues around the ward. Thus, while the frequency of verbal checks remained low, the checklist was an effective memory tool that reminded the porters to check for infection control requirements prior to transferring a patient.

Many studies have reported low adherence rate to checklists by medical workers. Healthcare professionals have largely resisted using checklists, dismissing them as “tick-box medicine” [26], or an insult to their intelligence [27]. An attempt to introduce a WHO surgical checklist in one UK institution reported a compliance rate of only 42% [28]. In spite of this, the study reported a noticeable improvement in safety processes such as timely use of prophylactic antibiotics, which rose from 57% to 77% of operations. Similar results were reported in the introduction of preventive care checklist [29] and anaesthesia checklist [30].

Our results are consistent with these previous studies of checklists. Negative attitudes towards the use of checklists are a major barrier to the implementation. Regulation of checklist use in healthcare is likely to be difficult to achieve. A fundamental change in culture and attitudes toward checklists is necessary [31]. The support of local champions, particularly among senior consultants, is critical to their success [32].

### Effectiveness of the coloured cue

The observed improvement in compliance with infection control precautions when a coloured cue was present is consistent with existing evidence on the importance of colour in the communication of information [33]. When a red sticker was attached to the transfer form, the information was noticed by the subjects immediately. The appropriate use of colour in safety signs is addressed under a series of standards published by the American National Standards Institute (ANSI) [34,35]. The choice of colour red to signify infectious cases was consistent with colour stereotypes used for communicating hazard. Informal interviews with the porters revealed general consensus on the effectiveness of the intervention. Some porters commented on their struggle to read information in the transfer form, due to diminished vision caused by presbyopia. Redundantly coding critical information with colours increased its detectability.

### Checklist versus coloured cue: which intervention is better?

Implementation of a pre-transfer checklist and coloured cue resulted in comparable improvement in compliance. The improvement gained from applying both interventions concurrently was only marginal, indicating that there were little additive benefits in implementing both interventions. Based on the reactions of the participants towards the intervention, however, it would appear that the coloured cue will be more effective in the longer term. As with any patient safety improvement strategies, acceptance by care providers is crucial to their success.

### Barriers to compliance with infection control precautions

While improved communication can enhance adherence to infection control precautions, however this alone is not sufficient in tackling this complex problem. Behavioural change remains a challenging obstacle. Infection control protocols were sometimes knowingly violated by the porters. Non-adherence to infection control precautions was also prevalent among nurses and physicians. Poor examples shown by superiors, and normalization of deviance can cultivate a culture of non-compliance in all parts of the hospital [36,37].

Another significant barrier to compliance was understaffing on the ward. When resources were stretched, the need to assist porters with transfer was a source of unwelcomed distraction. Transfer activities were often rushed, and infection control precautions were overlooked as a result. The role of understaffing in the spread of hospital acquired infection has been documented in several studies [38,39]. It might be conjectured that implementing the pre-transfer checklist further competed for the nurses’ attention, and this may have contributed to the low acceptability of the intervention.

Our observations further showed that there was variability in the performance of the required precautions. The guidelines for the use of gloves and gowns (as outlined in Table 1) were rarely adhered to. In particular, gloves worn when handling patients requiring contact precautions were not discarded or changed before touching environmental item and surfaces; gowns worn were not removed before leaving the patient’s room. The practice of hand antisepsis was particularly poor. The porters, in general, preferred using gloves as a protective barrier. There appeared to be a misconception that glove use eliminates the need for additional hand antisepsis. Hand antisepsis was almost never practised whenever gloves were used. This observation is consistent with existing evidence that the use of gloves represents a major barrier for compliance with hand antisepsis [40]. There is therefore a need to educate the porters on the importance of hand antisepsis, as the use of gloves cannot be guaranteed to provide complete protection against contamination of the hands.

Informal interviews with the porters and ward nurses also revealed that guidelines on infection control appeared to vary across different departments. In particular, there was confusion regarding whether a gown should be worn. Whilst standard protocol mandated the use of a gown when transporting patients requiring contact precautions [21], some staff members believed that wearing of gowns increased the risk of infection. Such conflicts in protocols led to confusion and frustration amongst ward nurses and porters. Clear and consistent guidelines need to be in place across the hospital.

Our observations confirm existing evidence that isolation precaution signage and promotional materials are ineffective in encouraging compliance. In the study site, posters were used to signal isolation precaution, and educational materials were displayed on the ward notice boards. However, compliance remained low. A review of the literature specifically exploring the impact of posters and promotional materials found little evidence that the messages they convey increase lasting compliance [41]. In most cases, compliance improved initially, but returned to baseline levels within several months.

### Limitations and future research

Our study had several limitations. Firstly, we evaluated the interventions at one hospital and the number of participants involved in the study was relatively small. Thus, our results may not be generalisable across settings. Secondly, there is the possibility of carry-over effects, where earlier exposure to one intervention affects a subject’s response when a different intervention is applied. To minimise carry-over effects, the interventions were randomised. Our observations showed that the rate of compliance for each study arm remained relatively stable throughout the experimental period, indicating that any carry-over effects were minimal. Finally, there is the possibility of Hawthorne effects, where subjects improve their compliance when being observed. Since the interventions were presented to the subjects by the researcher, and the researcher was present during the transfer, the subjects may have modified their compliance behaviour. To prevent this, the design of the interventions included the escort requirement so as to blind the subjects to the true intent of the study. To further minimise Hawthorne effects, the researcher shadowed transfers of both infectious and non-infectious patients.

In this study, we evaluated our interventions by measuring compliance with infection control precautions. Future studies should assess the effects of improved communication on actual infection rates. Further, the interventions were evaluated on a small number of healthcare personnel, over a short period of time. Follow-up studies should be carried out to examine if the effects of the interventions can be sustained over time on a larger sample size. The effectiveness of the interventions should also be evaluated in the absence of an observer. And finally, in this study, no specific efforts were made to encourage the use of the checklist. It is not clear whether active promotion would lead to improved compliance, but the hypothesis is certainly worthy of further investigation.

## Conclusions

Patients are routinely transported from one department to another during hospitalization. Ineffective communication of a patient’s infectious status can result in non-compliance with infection control precautions, exposing both staff members and other patients to the risk of infection. In this study, we demonstrated that simple measures to improve communication through the provision of a checklist and the use of coloured cue brought can potentially enhance compliance with infection control precautions. The study highlights the importance of communicating infection control information when patient care is transferred from one provider to another. To date, little research has been done to address this issue. Further investigations should be carried out to understand how infection control information can be more effectively communicated during transitions of care.

## Competing interests

The authors declare that they have no competing interests.

## Authors’ contributions

MSO designed the study, collected the data, conducted the data analysis and drafted the manuscript. EC and FM contributed to the design of the study, assisted in the interpretation of the data, and revised the manuscript. JP, SM, JW, WW and RC assisted with the coordination of the study, and revised the manuscript. All authors read and approved the final manuscript.

## Pre-publication history

The pre-publication history for this paper can be accessed here:

http://www.biomedcentral.com/1471-2334/13/72/prepub

## Supplementary Material

Additional file 1 **Observational tool.** Structured form used for data collection. Click here for file
