# Supplementary material for: Communication interventions to improve adherence to infection control precautions: a randomised crossover trial
Source: BMC Infect Dis. 2013 Feb 6;13:72. doi: 10.1186/1471-2334-13-72 (PMC3599084; doi:10.1186/1471-2334-13-72)
Supplement: Additional file 1 — Observational tool. Structured form used for data collection. [file 1471-2334-13-72-S1.pdf]

Observational Tool

Date: Porter:

Ward:

Interventions: Checklist Coloured Cue Both Control

Patient's infectious status: Contact precautions Non-infectious

Timing of transfer:

Porter departed Radiology:

Porter arrived at ward:

Ported departed ward with patient:

Porter arrived at Radiology with patient:

Communication of infection control precautions:

At Radiology prior to patient collection: Y N

At the ward on patient collection: Y N

Transfer form completed (Radiology): Y N

Transfer form completed (ward): Y N

Precautions taken by porter: Hand hygiene Gloves Gown None

Adherence to checklist: Y N

Descriptive data:
